# Analyzing the Relationship Between IOL Fixation and PCO Prevention

**DOI:** 10.3390/jcm14196947

**Published:** 2025-09-30

**Authors:** David Beckers, Florian Kretz, Jodhbir Mehta, Lena Beckers

**Affiliations:** 1 Precise Vision Ophthalmologists, 48 429 Rheine, Germany; f.kretz@precisevision.de (F.K.); dr.lenabeckers@gmail.com (L.B.); 2 Singapore National Eye Centre, Singapore 168751, Singapore; jodmehta@gmail.com

**Keywords:** YAG, PCO, capsulotomy, rhexis-fixation, cataract surgery

## Abstract

**Background**: Posterior capsular opacification (PCO) remains the most common long-term complication following cataract surgery. This correspondence investigates whether intraocular lens (IOL) fixation type influences PCO risk by comparing Nd:YAG capsulotomy rates between capsulotomy-fixated (FEMTIS) and conventional in-the-bag IOLs with similar material and edge profiles. **Methods**: A systematic review was conducted. Eligible studies reporting quantitative YAG rates at ≥3 months of follow-up were included. **Results**: FEMTIS IOLs demonstrated lower capsulotomy rates (3.1% at 12 months) compared to in-the-bag LENTIS lenses (4.7% at 12 months), despite identical optic designs and identical material. **Conclusions**: This suggests that capsulotomy fixation may promote closer capsule–optic contact and reduce the potential space for lens epithelial cell migration. While data are limited by study heterogeneity and follow-up duration, early evidence supports anterior fixation as a potential strategy to reduce PCO risk and improve long-term capsular clarity. Further prospective studies are warranted. Limitations include heterogeneous study designs, relatively short follow-up, and reliance on Nd:YAG capsulotomy as the sole endpoint.

## 1. Introduction

Cataract surgery is among the most commonly performed and successful surgical procedures worldwide, with intraocular lens (IOL) implantation restoring vision and quality of life for millions of patients [1]. Despite significant advances in surgical technique, IOL design and perioperative care, posterior capsular opacification (PCO) remains the most common long-term complication following cataract surgery [2]. In settings with limited access to follow-up care, untreated PCO can result in persistent visual impairment [3].

Beyond its clinical implications, posterior capsular opacification carries significant socioeconomic consequences. Nd:YAG capsulotomy, although effective, is not without drawbacks—it increases healthcare costs, requires specialized equipment, and is associated with potential complications such as retinal detachment, cystoid macular edema, and IOL damage. These risks highlight the importance of preventing PCO rather than merely treating it once established. Furthermore, with the rising demand for premium IOLs and patient expectations of spectacle independence, even subtle posterior capsule changes can compromise visual quality, contrast sensitivity, and satisfaction. This has driven innovation in IOL biomaterials, edge designs, and fixation concepts, yet no single approach has completely eliminated PCO. In this context, evaluating whether fixation site itself—independent of optic design or material—may alter capsular behavior represents an important step toward optimizing long-term outcomes in cataract surgery.

Recent systematic reviews and experimental studies underline that PCO prevention is multifactorial, spanning IOL material, optic edge profile, surgical technique, and emerging pharmacologic or drug-eluting strategies [4,5]. While each approach contributes incremental benefit, none has provided a definitive solution, underscoring the need to explore additional factors such as capsular fixation site.

PCO is caused by residual lens epithelial cells (LECs) that proliferate and migrate across the posterior capsule [6]. Various factors influence its development, including patient age, surgical technique, and IOL design [7]. A critical determinant is the proximity of the IOL optic to the posterior capsule, which can create a mechanical barrier to LEC migration. Greater optic–capsule contact typically reduces the risk of PCO, while a persistent space may facilitate cellular proliferation [7].

Conventional in-the-bag IOLs help stabilize the capsular bag through haptic tension, maintaining separation between the anterior and posterior capsules. However, this configuration may inadvertently preserve a potential space that enables LEC activity [5]. In contrast, capsulotomy-fixated lenses eliminate the need for haptic bag expansion and instead anchor the optic directly to the anterior capsule. This anterior fixation reduces bag volume and may promote closer optic–posterior capsule apposition, potentially mitigating the anatomical conditions conducive to PCO.

This correspondence takes a systemic review approach to compare published YAG capsulotomy rates between the capsulotomy-fixated and traditional in-the-bag designs, with similar optic material and edge profiles. We propose a physiological rationale for the observed reduction in PCO following anterior capsulotomy fixation and examine how this design feature may influence long-term capsular clarity.

## 2. Materials and Methods

A systematic review was conducted to compare published Nd:YAG capsulotomy rates following implantation of the FEMTIS (Teleon surgical, Spankeren, The Netherlands) capsulotomy-fixated intraocular lens (IOL) versus conventional in-the-bag IOLs with similar material and edge designs. The review followed PRISMA guidelines where applicable to correspondence format constraints.

A structured literature search was performed in PubMed and Embase from inception to April 2025 using the terms: (“FEMTIS” OR “capsulotomy-fixated IOL”) AND (“posterior capsular opacification” OR “PCO” OR “Nd:YAG capsulotomy”), and (“Lentis” OR “hydrophilic acrylic IOL”) AND (“in-the-bag”) AND (“posterior capsule opacification” OR “PCO” OR “YAG capsulotomy”).

To ensure comparability between studies, particular attention was given to IOL characteristics, as differences in optic edge design, material composition, or surface properties are known confounders in PCO research. By restricting inclusion to lenses from the same manufacturer (hydrophilic acrylic LENTIS and FEMTIS platforms) and identical square-edge optics, the analysis aimed to isolate fixation site as the primary variable. Study quality was assessed by reviewing sample size, follow-up duration, and clarity of outcome definitions.

In addition, the outcome of Nd:YAG capsulotomy was chosen as it represents a standardized, clinically relevant endpoint that reflects both the incidence and the functional impact of PCO on patients. While subjective grading systems exist, the capsulotomy rate provides an objective and reproducible measure across different study settings. However, reliance on Nd:YAG capsulotomy rates alone may not fully capture the clinical spectrum of PCO, as treatment timing can be influenced by patient expectations, lens type, or physician preference.

Eligible studies reported quantitative Nd:YAG capsulotomy rates following cataract surgery with either the capsulotomy-fixated FEMTIS IOL or conventional in-the-bag Lentis IOLs). Studies were included if they had ≥3 months of follow-up, clearly defined IOL model and fixation type, and sample size ≥ 50 eyes. Case reports, reviews, and studies with mixed IOL types or unclear data were excluded.

From each included study, the following were extracted: IOL model, fixation method, optic material, edge design, duration of follow-up, number of eyes, and cumulative Nd:YAG capsulotomy rate. Data were synthesized descriptively due to heterogeneity in follow-up duration and outcome reporting. Comparisons focused on capsulotomy incidence at comparable time points emphasizing studies using lenses from the same manufacturer to isolate the impact of fixation site.

## 3. Results

The YAG capsulotomy rate results reported by Stylianides et al. present a robust sample size and extended follow-up period. With data from 4388 eyes and a median follow-up of 4.2 years (extending up to 6 years), the study offers a reliable assessment of the hydrophilic LENTIS platform’s long-term performance in relation to posterior capsule opacification (PCO). The reported capsulotomy rates—4.7% at 1 year, 8.2% at 2 years, 17.2% at 4 years, and 22.4% at 6 years—are comparatively low for a hydrophilic acrylic IOL [7]. This large dataset, drawn from real-world clinical practice and strengthened by a systematic multi-step follow-up process, provides valuable insights into the Nd:YAG capsulotomy rate of the LENTIS platform, which is composed of the same hydrophilic acrylic material as the FEMTIS IOL.

When compared to the prospective evaluation by Auffarth et al., which reported a Nd:YAG capsulotomy rate of 3.1% at 12 months in 336 FEMTIS eyes [8], the Stylianides cohort showed a slightly higher 1-year rate of 4.7%. The absolute risk difference was 1.6% (95% CI −0.5% to 3.7%), and the relative risk was 1.52 (95% CI 0.74–3.14). While not statistically significant, the direction of effect suggests a trend toward lower Nd:YAG rates in the capsulotomy-fixated FEMTIS design.

In contrast, Kim et al. evaluated 913 eyes implanted with a refractive multifocal LENTIS platform and reported a Nd:YAG capsulotomy rate of 10.3% at ≥3 months [9]. Compared to the 3.1% 12-month rate reported by Auffarth et al., this corresponded to an absolute risk difference of 7.2% (95% CI 4.8–9.6%) and a relative risk of 3.32 (95% CI 1.72–6.42) (Table 1). This indicates a significantly higher early Nd:YAG rate in the multifocal LENTIS group. However, this finding must be interpreted in light of the known tendency for multifocal IOL recipients to undergo earlier capsulotomy due to higher visual demands and expectations [10], which may confound direct comparisons.

Taken together, these findings suggest that capsulotomy-fixated lenses may reduce the risk of early clinically significant PCO when compared to conventional monofocal in-the-bag designs of identical material and edge profile. However, higher Nd:YAG rates observed in multifocal designs likely reflect patient-related factors and threshold differences rather than purely material or fixation effects.

## 4. Discussion

The lower PCO and Nd:YAG capsulotomy rates observed with the FEMTIS IOL, despite its hydrophilic material and anterior fixation, challenge the assumption that in-the-bag fixation is inherently superior for preventing secondary cataract. Notably, the FEMTIS shares its optic material, shape, and edge design with the in-the-bag Lentis platform. This allows for a focused evaluation of fixation site as an independent variable in PCO development.

**Figure 1:**
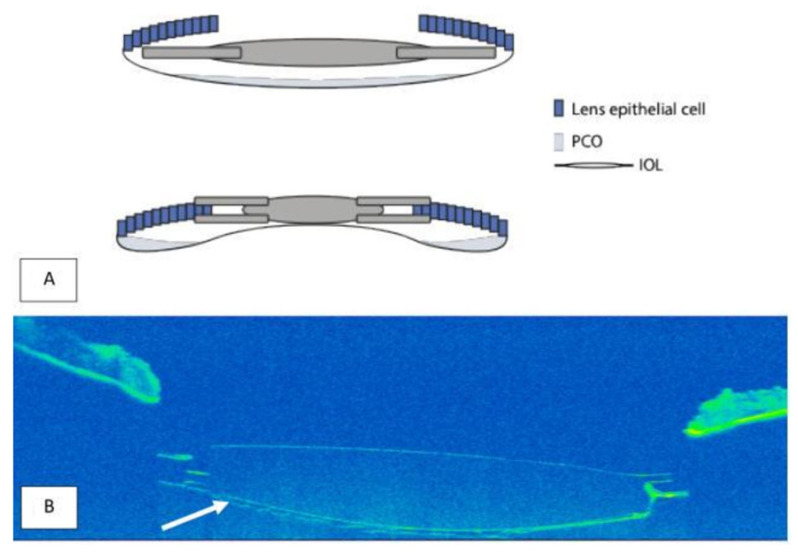
(**A**) Schematic illustration of lens epithelial cell (LEC) migration and posterior capsule opacification (PCO) formation after implantation of a standard in-the-bag IOL (top) versus a capsulorhexis-fixated IOL (bottom). (**B**) Anterior segment OCT image showing a capsulorhexis fixated IOL with a posteriorly attached posterior capsule (arrow).

In a large cohort of over 4000 eyes, Stylianides et al. reported YAG rates of 4.7% at one year and 22.4% at six years with the in-the-bag hydrophilic Lentis IOL. By comparison, Auffarth et al. [11] observed a 3.1% capsulotomy rate at 12 months with the FEMTIS platform, suggesting that anterior fixation may offer similar or even superior protection against early PCO despite the same material and edge features (Figure 1).

From a mechanistic perspective, the findings support the concept that the spatial relationship between the optic and posterior capsule is at least as critical as lens material or edge design in modulating PCO risk. By anchoring the optic to the anterior capsule, capsulotomy-fixated IOLs appear to reduce capsular bag volume and enforce a tighter capsule–optic interface, thereby limiting the scaffold available for lens epithelial cell migration. This raises considerations for IOL selection, particularly in younger patients or those at higher risk of PCO, where long-term capsular clarity is crucial for maintaining visual quality.

These findings suggest that anterior fixation reduces the space between the posterior capsule and the optic, minimizing the potential for LEC proliferation. This contrasts with conventional in-the-bag IOLs, which rely on outward haptic force to maintain capsular contour—possibly maintaining a scaffold for PCO to develop. While the precise centration enabled by femtosecond laser-assisted capsulotomy in the FEMTIS system may reduce capsular folds or edge gaps, several studies have reported a higher incidence or earlier need for Nd:YAG capsulotomy following femtosecond laser-assisted cataract surgery [9,10]. Other potential trade-offs of anterior fixation include increased anterior capsular fibrosis and challenges in IOL exchange.

Beyond anatomical considerations, patient-specific factors must also be acknowledged. Younger patients generally show higher PCO rates due to more active lens epithelial cells, while systemic conditions such as diabetes and uveitis can further accelerate capsular changes. Moreover, recipients of premium multifocal and toric IOLs may request Nd:YAG capsulotomy earlier due to subtle visual disturbances, potentially inflating capsulotomy statistics compared to monofocal lenses. Thus, the observed difference between FEMTIS and Lentis designs is particularly notable, as it persists even in contexts where patient expectations and comorbidities may vary.

The health-economic implications are also considerable. Nd:YAG capsulotomy, while routine in developed healthcare systems, is associated with costs, follow-up visits, and potential complications such as cystoid macular edema, retinal detachment, and IOL pitting. In regions with limited access to ophthalmic laser facilities, untreated PCO may lead to prolonged visual impairment, with direct effects on quality of life. By reducing the frequency of capsulotomy, capsulotomy-fixated lenses could therefore decrease the overall economic burden of cataract surgery and improve equity of care, especially in resource-limited settings.

Nonetheless, several limitations warrant consideration. Most published studies, including those cited here, involve relatively short-term follow-up. Additionally, direct comparative data remain sparse, and variability in surgical technique, patient selection, and follow-up protocols limits generalizability. Factors such as capsulorhexis size, capsular fibrosis patterns, and patient comorbidities may independently influence PCO formation and were not consistently controlled. Widespread adoption of capsulotomy-fixated designs will therefore require prospective randomized studies with extended follow-up to confirm durability of the observed effect and to monitor for potential trade-offs such as capsular fibrosis or difficulties in IOL exchange. Specifically, the current analysis is constrained by a limited evidence base, heterogeneous patient populations, and variable follow-up durations. Furthermore, surgeon technique, femtosecond laser–related variables, and patient selection factors were not consistently controlled and may have influenced outcomes. Potential trade-offs of anterior fixation, such as increased anterior capsular fibrosis, must also be considered.

Given the limited number and heterogeneity of available studies, our findings should be regarded as hypothesis-generating rather than definitive.

Looking ahead, the principle of fixation-site modification may open new perspectives for IOL design. Combining anterior capsulotomy fixation with advanced biomaterials, improved edge profiles, or surface modifications targeting lens epithelial cell adhesion could provide a multifaceted strategy for further reducing PCO.

These insights underscore the need to refine fixation concepts alongside ongoing innovations in IOL optics and biomaterials to achieve more complete prevention of secondary cataract.

## 5. Conclusions

In conclusion, clinical data suggest that capsulotomy-fixated IOLs like the FEMTIS may reduce PCO through a distinct anatomical mechanism—by eliminating the potential space for LEC migration and promoting close capsule–optic contact. These results support the continued investigation of anterior fixation as a strategy to improve long-term capsular clarity. Importantly, the concept challenges the conventional paradigm that in-the-bag fixation is the gold standard for minimizing secondary cataract, highlighting that fixation site itself may represent an underexplored determinant of PCO prevention. For surgeons, this raises the prospect of tailoring IOL choice not only according to refractive goals and material properties but also on the basis of long-term capsular behavior. Future prospective, controlled studies with extended follow-up are needed to validate these observations, assess their generalizability across diverse patient populations, and clarify whether anterior fixation can provide durable advantages in real-world practice. If confirmed, such findings could influence future IOL design strategies and contribute to reducing the global burden of Nd:YAG capsulotomy.

## Figures and Tables

**Table 1:** Capsulotomy rate with comparable IOL material.

Study/Author	Sample Size	Follow-Up	IOL Type	YAG Capsulotomy Rate
Stylianides et al. [7]	4388 eyes	Median 4.2 years (up to 6)	Hydrophilic LENTIS platform—monofocal	1 yr: 4.7% 2 yr: 8.2% 4 yr: 17.2% 6 yr: 22.4%
Auffarth et al. [11]	336 eyes	12 months	Hydrophilic FEMTIS platform	12 mo: 3.1%
Kim et al. [9]	913 eyes	≥3 months	Hydrophilic LENTIS platform—Refractive multifocal IOL	≥3 mo: 10.3%

## Data Availability

The data that support the findings of this study are available from the corresponding author upon request.
